# Single-case design meta-analyses in education and psychology: a systematic review of methodology

**DOI:** 10.3389/frma.2023.1190362

**Published:** 2023-11-13

**Authors:** Mariola Moeyaert, Marzieh Dehghan-Chaleshtori, Xinyun Xu, Panpan Yang

**Affiliations:** ^1^Department of Educational and Counseling Psychology, University at Albany-State University of New York, Albany, NY, United States; ^2^Center of Tsinghua Think Tanks, Tsinghua University, Beijing, China; ^3^Center for Research on Child Wellbeing, Princeton University, Wallace Hall, Princeton, NJ, United States

**Keywords:** Single-Case Experimental Design, meta-analysis, Monte Carlo simulation, methodological research, systematic review

## Abstract

Meta-analysis is of increasing importance as this quantitative synthesis technique has the potential to summarize a tremendous amount of research evidence, which can help making evidence-based decisions in policy, practice, and theory. This paper examines the single-case meta-analyses within the Education and Psychology fields. The amount of methodological studies related to the meta-analysis of Single-Case Experimental Designs (SCEDs) is increasing rapidly, especially in these fields. This underscores the necessity of a succinct summary to help methodologists identify areas for further development in Education and Psychology research. It also aids applied researchers and research synthesists in discerning when to use meta-analytic techniques for SCED studies based on criteria such as bias, mean squared error, 95% confidence intervals, Type I error rates, and statistical power. Based on the summary of empirical evidence from 18 reports identified through a systematic search procedure, information related to meta-analytic techniques, data generation and analysis models, design conditions, statistical properties, conditions under which the meta-analytic technique is appropriate, and the study purpose(s) were extracted. The results indicate that three-level hierarchical linear modeling is the most empirically validated SCED meta-analytic technique, and parameter bias is the most prominent statistical property investigated. A large number of primary studies (more than 30) and at least 20 measurement occasions per participant are recommended for usage of SCED meta-analysis in Education and Psychology fields.

## Introduction

Single-Case Experimental Designs (SCEDs) are experimental designs used to repeatedly measure one or multiple participants across at least two conditions: a condition without intervention (i.e., baseline condition) and a condition with intervention (i.e., intervention or treatment condition). Intervention effectiveness can be evaluated by comparing outcome data obtained from each participant's baseline condition with subsequent intervention condition. SCEDs are strong experimental designs because the individual participant provides their own control condition for comparison purposes. The main goal of comparing outcome data under these conditions is to determine whether there is a causal relationship between the introduction of the intervention and changes in outcome data (Onghena, 2005). This functional relationship is participant-specific and cannot be generalized beyond the participant (Van den Noortgate and Onghena, 2003a,b). To increase the external validity of intervention effectiveness, SCED studies traditionally include multiple participants (e.g., Caron and Dozier, 2019; Petrocchi et al., 2021). Indeed, Shadish and Sullivan (2011) found an average number of study participants of 3.64, indicating multiple participants per SCED study. A more recent systematic review by Jamshidi et al. (2020) confirmed this finding and indicated an average of four participants per SCED study. Thus, outcome data obtained through SCED studies are often ordered according to a two-level hierarchical structure: repeated outcome data (i.e., level 1) nested within participants (i.e., level 2). To further generalize intervention effectiveness and contribute to evidence-based practices, summarizing evidence from one SCED study is insufficient. Therefore, several researchers have suggested combining evidence across SCED studies meeting strict inclusion criteria using meta-analytic techniques (i.e., Van den Noortgate and Onghena, 2008; Moeyaert et al., 2013a; Shadish et al., 2013 and, e.g., Asaro-Saddler et al., 2021). This results in outcome data structured according to three levels: repeated outcome data (i.e., level 1) are nested within participants (i.e., level 2), and participants, in turn, are nested within studies (i.e., level 3).

### Meta-analytic techniques for SCEDs in education and psychology fields

Two meta-analytic techniques for synthesizing SCED studies were previously empirically validated in published methodological research: multilevel modeling (MLM) and weighted average of summary statistics. Both are discussed in the following sections.

#### Multilevel modeling

The multilevel modeling (MLM) framework is promising for combining SCED data across participants and across studies as it takes the natural hierarchical data structure (and the dependencies derived from it) into account (Borenstein et al., 2009): outcome data are clustered within participants, and participants, in turn, are clustered within studies. The hierarchical linear model (HLM), as an extension of the piecewise regression equation (Center et al., 1985), has been empirically investigated through multiple Monte Carlo simulation studies and is the most popular SCED meta-analytic technique. For example, Van den Noortgate and Onghena (2003a,b) proposed and validated two-level HLM to combine SCED data across participants. In 2007 and 2008, they extended the two-level HLM to three-level HLM to combine SCED data across studies (Van den Noortgate and Onghena, 2007, 2008). Later, Moeyaert et al. (2013a,b), Moeyaert et al. (2014) and Ugille et al. (2012, 2014) empirically validated three-level HLM for SCED meta-analysis using large-scale Monte Carlo simulation methods.

The HLM meta-analytic model can be used to estimate the overall effect size(s) across participants and studies (i.e., fixed effects) and study-specific and participant-specific deviations from the overall average effect size (i.e., random effects, see Moeyaert, 2019). Ever since Van den Noortgate and Onghena (2003a,b) proposed the usage of HLM for meta-analysis of SCED data, its statistical properties have been intensively investigated and validated through Monte Carlo simulation studies (e.g., Ugille et al., 2012; Moeyaert et al., 2013a,b, 2014; Declercq et al., 2020). It also has been applied in many SCED meta-analyses (e.g., Asaro-Saddler et al., 2021; Fingerhut and Moeyaert, 2022).

The most often seen models for the three-level HLM are the models with two parameters and with four parameters. In the two parameters HLM approach, a dummy variable indicating the phase (i.e., 0 for the baseline phase and 1 for the intervention phase) is the sole independent variable. This model provides an estimate of the baseline level and the level change between the baseline and intervention phases. The four parameters HLM approach, on the other hand, often includes the following four independent variables: phase, time (i.e., session number), and the interaction term between phase and time. This results in an estimate of the level at the start of the baseline, the baseline trend, and the level and trend change between the baseline and intervention phases. Additional parameters can be added to the four parameter HLM approach. For instance, two parameters can be added to model quadratic time trends: time squared and the interaction term between phase and time squared. The six parameters HLM approach has been studied infrequently.

##### Three-level HLM with two parameters

The two parameters three-level HLM approach is most frequently studied and is represented through Equations 1 to 3:


(1) $$\begin{array}{l} \text{Level 1 (observation-level)}:y_{ijk}=\beta_{0jk}+\beta_{1jk}Phase_{ijk}+e_{ijk} \end{array}$$

(2) $$\begin{array}{l} \text{Level 2 (participant-level)}:\left\{\begin{array}{l} \beta_{0jk}=\theta_{00k}+u_{0jk}\\ \beta_{1jk}=\theta_{10k}+u_{1jk} \end{array}\right\} \end{array}$$

(3) $$\text{Level 3 (study-level)}:\left\{\begin{array}{l} \theta_{00k}=\gamma_{000}+v_{00k}\\ \theta_{10k}=\gamma_{100}+v_{10k} \end{array}\right\}$$

Equations (1)–(3) represent the first level (i.e., observation level), the second level (i.e., participant level), and the third level (i.e., study level) of the HLM framework, respectively. The SCED meta-analysis includes *K* number of studies and *J* number of participants, and each participant has been repeatedly measured over a total of *I* number of time points. *y*_*ijk*_ is the outcome value at observation session *i* (*i* = 1, 2, ..., *I*) of participant *j* (*j* = 1, 2, …, *J*) in study *k* (*k* = 1, 2, …, *K*). *Phase* is a dummy coded variable with 0 representing the baseline phase and 1 representing the intervention phase. Equation (1) is a regression equation at the observation level that uses *phase* to model outcome *y*_*ijk*_. Thus, β_0*jk*_ indicates the baseline level of participant *j* in study *k*, and β_1*jk*_ represents the level change between the baseline and intervention phases of participant *j* in study *k*. Another symbol in Equation (1) is *e*_*ijk*_ representing the within-participant residual standard deviation of participant *j* (*j* = 1, 2, …, *J*) at observation session *i* (*i* = 1, 2, …, *I*) in study *k*.

Equation (2) is the participant-level equation indicating that the baseline level of participant *j* (i.e., β_0*jk*_) can be obtained by the average baseline level (i.e., θ_00*k*_) across all participants from study k and the deviation of participant j from this study average (i.e., *u*_0*jk*_). Similarly, the level change of participant *j* (i.e., β_*ijk*_) equals the sum of the average level change (i.e., θ_10*k*_) across *J* participants from study k and the deviation of participant *j* (i.e., *u*_1*jk*_) from this study *average*. The deviations at level 2 are assumed to be normally distributed with a variance of $\sigma_{u_{0}}^{2}$ (i.e., between-participant variance in baseline level) and $\sigma_{u_{1}}^{2}$(i.e., between-participant variance in intervention effect).

In Equation (3), γ_000_ represents the average baseline level across *K* studies, and *v*_00*k*_ indicates the between-study deviation of the baseline level. Likewise, γ_100_ represents the average level change across *K* studies, and *v*_10*k*_ indicates the between-study deviation of the level change. The deviations at level 3 are assumed to be normally distributed with a variance of $\sigma_{v_{0}}^{2}$ (i.e., between-study variance in baseline level) and $\sigma_{v_{1}}^{2}$(i.e., between-study variance in intervention effect).

##### Three-level HLM model with four parameters

The two parameters HLM approach can be expanded to four parameters HLM by adding two independent variables (i.e., time and the interaction term between time and phase):

Level 1 (observation-level):


(4) $$\begin{array}{l} y_{ijk}=\beta_{0jk}+\beta_{1jk}Phase_{ijk}+\beta_{2jk}{Time0}_{ijk}+\beta_{3jk}PhaseTimeC_{ijk}\\ +e_{ijk} \end{array}$$

(5) $$\begin{array}{l} \text{Level 2 (participant-level)}:\left\{\begin{array}{l} \beta_{0jk}=\theta_{00k}+u_{0jk}\\ \beta_{1jk}=\theta_{10k}+u_{1jk}\\ \beta_{2jk}=\theta_{20k}+u_{2jk}\\ \beta_{3jk}=\theta_{30k}+u_{3jk} \end{array}\right\} \end{array}$$

(6) $$\begin{array}{l} \text{Level 3 (study-level)}:\left\{\begin{array}{l} \theta_{00k}=\gamma_{000}+v_{00k}\\ \theta_{10k}=\gamma_{100}+v_{10k}\\ \theta_{20k}=\gamma_{200}+v_{20k}\\ \theta_{30k}=\gamma_{300}+v_{30k} \end{array}\right\} \end{array}$$

Equations (4)–(6) represent the first level (i.e., observation level), the second level (i.e., participant level), and the third level (i.e., study level) of the HLM, respectively. Compared to the two parameters HLM approach, the four parameters HLM approach includes two additional independent variables: Time0 and PhaseTimeC. Time0 is a time-related continuous variable that indicates the sequence in measuring the outcomes. Time0 equals zero for the first observation session. TimeC is also a time-related continuous variable, but it is centered around the beginning of the intervention phase. PhaseTimeC is an interaction term between TimeC and Phase. With these two additional parameters, this four parameters HLM approach can estimate the outcome level at the start of the baseline phase, baseline trend, outcome level change between the baseline and intervention phases at the start of the intervention, and trend change between baseline and intervention phases. In Equation (4), β_2*jk*_ and β_3*jk*_ represent the baseline trend and trend change between the baseline and intervention phase for participant *j* from study *k*. θ_20*k*_ and θ_30*k*_ represent the average baseline trend and the trend change across *J* participants in study *k*. γ_200_ and γ_300_ represent the average baseline trend and the trend change across *K* studies. *u*_2*jk*_ and *u*_3*jk*_ are the between-participant deviations of baseline trend and trend change of participant *j* in study *k* (assumed to be normally distributed with variance $\sigma_{u_{2}}^{2}$and $\sigma_{u_{3}}^{2}$, respectively). *v*_20*k*_ and *v*_30*k*_ are the between-study deviations of baseline trend and trend change in study *k* (assumed to be normally distributed with variance $\sigma_{v_{2}}^{2}$and $\sigma_{v_{3}}^{2}$, respectively).

##### Three-level HLM model with six parameters

The six parameters model includes the same parameters as in Equations (4)–(6) with two additional ones: Time squared (i.e., Time2) and the interaction term between Phase and Time squared (PhaseTime2), see Equation (7):


(7) $$\begin{array}{l} y_{ijk}=\beta_{0jk}+\beta_{1jk}Time_{ijk}+\beta_{2jk}Time2_{ijk}+\beta_{3jk}Phase_{ijk}\\ \;+\beta_{4jk}Phase_{ijk}Time_{ijk}+\beta_{5jk}Phase_{ijk}Time2_{ijk}+e_{ijk} \end{array}$$

With these two additional parameters, this six parameters HLM approach can estimate the outcome level at the start of the baseline phase, baseline linear trend, baseline quadratic trend, outcome level change between baseline and intervention phases at the start of the intervention, linear trend change and quadratic trend change between baseline and intervention phases. In Equation (7), β_2*jk*_ and β_5*jk*_ represent the baseline quadratic trend and quadratic trend change between the baseline and intervention phase for participant *j* from study *k*. Similar to the previous approaches, the level 1 parameters vary at levels 2 and 3, and similar interpretations can be made.

The three aforementioned models (i.e., three-level HLM with two, four, and six parameters) are suitable for replicated AB designs and multiple baseline designs (MBDs). The models can be modified to meta-analyze data from ABAB withdrawal designs, alternating treatment designs (ATD), and other SCED designs (see Moeyaert et al., 2015, 2022). In addition, moderators can be added to explain the variance when there is between-participant variance and/or between-study variance in parameter estimates. More information about moderators in SCEDs meta-analysis using HLM can be found in Moeyaert et al. (2021, 2022).

#### Average of summary statistics

For each study participant, an effect size (e.g., the difference in means, non-overlap statistic, or regression-based statistic) can be calculated (i.e., summary statistic). Next, a simple average or weighted average of these participant-specific summary statistics can be estimated using the simple average or multilevel modeling techniques. Both results in an estimate of the overall average intervention effect. Because effect sizes from participants nested within one study are dependent, it is recommended to use cluster-robust variance estimation (RVE) in combination with calculating the simple average or multilevel modeling (see Chen and Pustejovsky, 2022). Because RVE is new to SCED meta-analysis, no methodological publications validating this approach were identified in the current systematic review.

### Data analysis estimation methods in SCED meta-analyses in education and psychology fields

When a model is selected, there are many ways to identify the best-fit statistic value as the estimate of the parameters. The data analysis estimation methods include but are not limited to maximum likelihood estimation (MLE), least square estimation (LSE), and Bayesian analysis. MLE is a likelihood-based estimation approach that is frequently used for parameter estimation (Rossi, 2018). MLE finds the value with the highest likelihood based on the data under the assumed probability distribution (Pan and Fang, 2002; Myung, 2003). Restrict maximum likelihood estimation (RMLE) is a variation of MLE that uses a likelihood function to calculate the best-fit estimate instead of identifying the highest likelihood estimate based on the data. LSE is also widely used to estimate parameters. It identifies the best-fit statistic value by minimizing the squared discrepancies between the observed and expected data (Rosenbaum et al., 2005; Everitt and Howell, 2021). There are various kinds of LSE, such as ordinary least squares (OLS) and weighted least squares (WLS). Both OLS and WLS are a type of generalized least squares (GLS). Another estimation method is Bayesian estimation. When using Bayesian estimation techniques, the researcher begins with the specification of one or more parameters, such as regression coefficients and variances or standard deviations or precisions (i.e., the inverse of variances) known as the prior distribution. This preliminary information is combined with the information in the data to create the posterior distribution. The posterior distribution captures the researcher's knowledge of the parameters at the end of the study (see Rindskopf, 2014, for a detailed demonstration of using Bayesian analysis for single-case designs).

### Current study

Previously, a few systematic reviews of SCED meta-analyses were conducted to summarize characteristics of content-related SCED meta-analyses (e.g., Moeyaert et al., 2021; Jamshidi et al., 2022). However, none of them focused on the methodology, and they excluded methodological papers in their reviews. Methodological work related to SCED meta-analytic techniques is largely overlooked; therefore, this study is timely and targets a broad audience. Applied SCED meta-analysts can learn about the available techniques that have been empirically investigated and the conditions under which the technique(s) is(are) suitable (given its desirable statistical properties). Methodologists can learn about the need for potential further methodological research and the commonly encountered realistic design conditions and parameter values included in previous methodological work. More specifically, this paper aims to address the following research questions:

1. What meta-analytic techniques, suitable to quantitatively synthesize SCED research, have been empirically validated through the Monte Carlo simulation?
2. What SCED meta-analytic data generation models were used in the Monte Carlo simulation studies?
3. What are the design conditions and parameter values included in the Monte Carlo simulation studies?
4. What statistical properties are investigated in the Monte Carlo simulation studies?
5. What are the conclusions regarding the statistical properties, per meta-analytic technique? In other words, under which realistic SCED meta-analytic conditions are investigated meta-analytic techniques recommended?
6. What is the purpose of each meta-analytic study?

## Methods

This systematic review was performed following the Preferred Reporting Items for Systematic Reviews and Meta-Analyses (PRISMA; Page et al., 2021) guideline. The PRISMA checklist is included as an Appendix.

### Eligibility criteria

The two specific inclusion criteria were: (1) methodological research, including Monte Carlo simulation or other validation techniques, and (2) the design is a SCED meta-analysis. The exclusion criteria were (1) designs other than SCED meta-analysis, (2) non-methodological research, (3) fields outside Education and Psychology, (4) non-human participants, and (5) non-English resources.

### Information sources

An extensive systematic literature search was conducted in December 2021 using the following databases: PsycINFO, Education Resources Information Center (ERIC), Web of Science (WOS), PubMed, DANS EASY (Archive hosting thousands of datasets), and ProQuest Dissertation and Thesis. PsycINFO, ERIC, and WOS were identified to have the most considerable scope within the field of psychology and education sciences. PubMed, DANS EASY, and ProQuest Dissertation and Thesis were used to search for gray literature.

### Search strategy

Two independent researchers conducted the systematic search. These two researchers were doctoral students trained in the field of single-case design and meta-analysis (and had taken graduate-level courses on both subjects and collaborated on a few other systematic review and meta-analysis projects).

The independent researchers used the search terms presented in Table 1 for each electronic database. These were modified to fit the search format for each database (see Appendix A for the syntax used in each database). Finally, the search terms were combined using the “OR” and “AND” Boolean operators. An interobserver agreement of 100% was obtained.

**Table 1:** Search strings electronic databases.

**Searched concepts anywhere**
1. “single case”
2. “single subject”
3. “N of 1”
4. “small N”
5. “multiple baseline”
6. “alternating treatment”
7. “reversal design”
8. “withdrawal design”
9. (1 OR 2 OR 3 OR 4 OR 5 OR 6 OR 7 OR 8)
10. “meta-analysis”
11. “synthesis”
12. “review”
13. (10 OR 11 OR 12)
14. “simulation”
15. “Monte Carlo”
16. “monte carlo”
17. (14 OR 15 OR 16)
18. (9 AND 13 AND 17)

Next, the title and abstract of the retained reports were downloaded, exported, and imported into Rayyan. Rayyan (http://rayyan.qcri.org, Quzzani et al., 2016) is a free Application that helps screen the studies and was used to de-duplicate and select the eligible reports.

After the first screening, the eligible reports were classified per journal. This was used to identify the top three journals publishing methodological research related to SCED meta-analyses: *Behavior Research Methods, Journal of Experimental Education*, and *Multivariate Behavioral Research*. The three journal websites were systematically searched using the same search strings (see Table 1; Appendix A) as the systematic search of electronic databases. Once the final pool of eligible studies was set, the two independent researchers completed the backward search. Finally, the references of all reports included in the final pool were searched, and there was 100% interobserver agreement that no extra studies met the eligibility criteria.

### Data collection

Every study was perused, and information about the meta-analytic technique, data generation model, design conditions, parameter values, statistical properties, conclusions and recommendations per meta-analytic technique, and research purpose were extracted.

The meta-analytic techniques in SCED were coded into four categories: (1) three-level hierarchical linear modeling (HLM, multilevel modeling, or linear mixed effects modeling), (2) generalized linear mixed effects modeling (GLMM), (3) meta-analysis of summary data (including the simple average of effect sizes, the median of effect sizes, and weighted average of effect sizes), and (4) other(s).

The data generation model was coded into three categories: (1) three-level HLM approach with two parameters (i.e., baseline level and level change between baseline and intervention phases), (2) three-Level HLM approach with four parameters (i.e., baseline level, baseline trend, level and trend change between baseline and intervention phases), and (3) other(s).

The data-generation design included information about the design of the primary SCED studies (e.g., MBD, AB), the number of primary SCED studies, the number of participants in each study, and the number of observation sessions.

The data analysis estimation method was coded into four categories: (1) MLE or REML, (2) OLS, (3) GLS, and (4) other(s).

The parameter values referred to the hypothesized values that researchers assigned for simulating SCED meta-analytic data. The type and number of parameters were based on the data-generation models.

Information about studied statistical properties was also retrieved. The statistical properties investigated can be defined as follow: the difference between the expected effect estimate and the true population effect (absolute bias); absolute bias divided by the population parameter value (relative bias; Moeyaert et al., 2014); the relative bias for the standard error parameter (relative SE bias); the proportion of 95% confidence intervals that contain the estimated parameter (95% confidence interval coverage; Owens, 2011); a measure of the average squared errors (mean squared error or MSE; Petit-Bois, 2014); the MSE divided by the squared nominal parameter value (relative MSE; Declercq et al., 2019); square root of mean of the squared difference of the estimated parameter from the true parameter (root mean square error or RMSE; Baek et al., 2020); the standard deviation of the sampling distribution of the effect estimator (standard error or SE; Moeyaert et al., 2014); the probability of rejecting the null hypothesis when the null hypothesis is true (Type I error); the probability of rejecting the null hypothesis when in fact a certain alternative parameter value is true (power; Cohen, 2013).

The research purpose was coded into six categories considering examination of the performance/appropriateness of a model or comparing the performances/appropriateness of models in terms of (1) statistical properties; (2) handling different SCED designs; (3) handling data complexity; (4) accounting for different effect size metrics; (5) handling model misspecification; and (6) others.

These items can be found in the codebook (see Appendix B) and were piloted by the same two independent researchers involved in the data selection. Six studies were randomly selected to be coded by two independent reviewers to calculate the IOA. The calculated IOA was 87%. After discussing and resolving the discrepancies, one coder proceeded with the coding of the remaining studies. Data extracted from included studies and used for the analysis can be accessed by contacting the corresponding author.

### Study selection

Through Rayyan, the two independent researchers reviewed the titles and abstracts and applied the inclusion and exclusion criteria. First, the researchers added labels reporting the reason for excluding reports. Then, based on the exclusion criteria, the following labels were predefined to select from (1) designs other than SCED meta-analysis, (2) non-methodological research, (3) studies reported in languages other than English, (4) fields outside education and psychology, and (5) non-human participants. The first screening (reviewing the titles and abstracts) was over-inclusive, as recommended by Higgins et al. (2021). For instance, titles and abstracts that did not contain enough information to determine eligibility were included. The same procedure was followed for the full-text screening.

**Figure 1:**
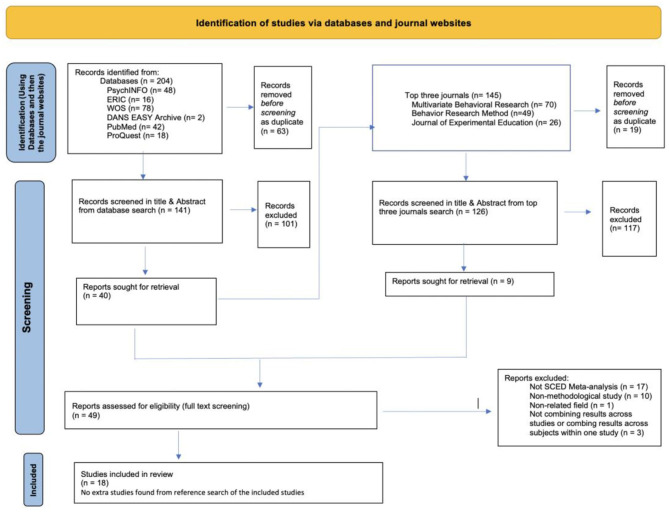
Flowchart of study selection.

The initial search yielded 204 results [PsychINFO (*n* = 48), ERIC (*n* = 16), WOS (*n* = 78), DANS EASY Archive (*n* = 2), PubMed (*n* = 42), and ProQuest Dissertation and Thesis (*n* = 18)]. For a more detailed overview, see the PRISMA flowchart displayed in Figure 1. All study references per database were imported into Rayyan (Quzzani et al., 2016). Using Rayyan, sixty-three duplicates were found, resulting in 141 remaining reports for screening. When the abstract screening was completed, 40 reports remained. The titles and abstracts screening was done by two reviewers with an IOA of 87%. Discrepancies were discussed and resolved between the two reviewers. The discrepancies were due to demonstration/illustration studies that were not looking into empirical validation of a meta-analytic model suitable for quantitative synthesis of SCED studies.

After the title and abstract screening was completed and discrepancies were solved, the top three journals (i.e., *Behavior Research Methods, Journal of Experimental Education*, and *Multivariate Behavioral Research*) were systematically searched using the same search terms. This search yielded 145 articles [*Multivariate Behavioral Journal* (*n* = 70), *Behavior Research Method* (*n* = 49), and *Journal of Experimental Research* (*n* = 26)]. After importing the articles in Rayyan and checking for duplicates, 126 new articles were identified. Out of the 126 articles, only nine met the inclusion criteria after title and abstract screening. The title and abstract screening at this stage was also conducted by the same two independent researchers, who reached 79.2% of IOA. The discrepancies were discussed and resolved afterward. Discrepancies were due to insufficient information in the abstract to decide if the resource met the inclusion criteria.

The full-text screening was accomplished by the same two independent researchers, and an IOA of 79.2% was obtained. Discrepancies were discussed and resolved. Discrepancies were because of demonstration/illustration studies that are not looking into empirical validation of a meta-analytic model suitable for quantitative synthesis of SCED studies.

After the full-text screening, the final pool of eligible reports contained 18 entries (both journal articles and dissertations). Later, the two independent reviewers went over the references of all 18 resources to do a citation search and found no additional articles meeting the inclusion criteria.

### Analysis

The raw data retrieved from the SCED meta-analysis methodological studies were saved and analyzed in Excel (Microsoft Corporation, 2018). Descriptive analyses were used to summarize the meta-analytic characteristics (i.e., meta-analytic technique, data generation model, data generation design, and data estimation) of the included 18 SCED meta-analysis methodology studies. Then, the design conditions and statistical properties were summarized using frequency tables.

Using data retrieved from these 18 reports, the next section contains a summary of the meta-analytic techniques, data generation models, data analysis models, and data generation designs. Next, the results related to design conditions, statistical properties, recommended conditions under which each meta-analytic technique is appropriate, and the research purpose of each study are synthesized.

## Results

### Meta-analytic techniques

An overview of meta-analytic characteristics, including meta-analytic technique and data generation model used in SCED meta-analysis methodological studies in Education and Psychology fields using HLM, is presented in Table 2 of Appendix C. HLM (or multilevel analysis, linear mixed-effects model) was the most frequently investigated SCED meta-analytic technique (i.e., *n* = 17, 94.4%). The other SCED meta-analytic technique, empirically validated, was the average of effect sizes, which was only used in one study (i.e., Idleman, 1993). Idleman (1993) compared three quantification metrics (i.e., PND, Glass' effect sizes, and Hedge's unbiased estimators) and used the average of the effect size as the method to aggregate the treatment effect.

### Data generation and data analysis models

The majority of the studies used one model to generate data, while two studies used multiple models to generate data (Idleman, 1993; Declercq et al., 2020). Among the 17 studies that used HLM as the meta-analysis technique, 14 (78%) used three-level HLM with four parameters, two (11%) used three-level HLM with two parameters (i.e., Owens, 2011; Moeyaert et al., 2016), and one used three-level HLM with two, four, and six parameters (i.e., Declercq et al., 2020) to generate data. The only study that used a meta-analysis of summary statistics (i.e., Idleman, 1993) generated normal or chi-squared distributed data with and without autocorrelation.

Most of the studies used the same analysis model as the data generation model to estimate the parameters of interest (specifically, about half of included studies: *n* = 8; 44.4%). Two studies (Declercq et al., 2020; Joo et al., 2021) used a slightly alternative analysis model, which allowed us to investigate the impact of model misspecification. Five studies (28%) used both the same analysis model and a slightly alternative analysis model. These studies are Moeyaert et al. (2013b), Petit-Bois (2014), Moeyaert et al. (2016), Petit-Bois et al. (2016), and Jamshidi et al. (2020). Three studies used completely different alternative analysis models. For example, Baek et al. (2020) did not specify the data generation model but used eight different analysis models (i.e., four three-level models and four four-level models). Manolov et al. (2014) used the three-level HLM with four parameters to simulate data. They used the weighted average of non-overlap statistics (e.g., NAP) and the standardized mean difference to estimate the overall average intervention effect. Similarly, Idleman (1993) simulated normal or chi-squared distributed data and analyzed the data using the average PND, average Glass' delta, and average Hedge's g.

### Data generation designs

Although multilevel models can be used to meta-analyze SCED data from a variety of designs, such as alternating-treatment designs (ATD), replicated ABAB designs, and changing criterion designs (Shadish et al., 2013; Jamshidi et al., 2022), the majority of reports generated data using a multiple baseline design (MBD; 78%; *n* = 14). This is not surprising as MBDs are the most frequently used designs in practice (Jamshidi et al., 2022) and are recognized as having higher internal and external validity than other SCED types (Baek et al., 2020; Jamshidi et al., 2022). Three studies (i.e., Idleman, 1993; Owens, 2011; Tsai, 2011) simulated basic AB design data (17%), and one study (i.e., Declercq et al., 2020) used replicated AB design data (5%).

### Data analysis estimation methods

The most popular estimation procedure was REML. Among the 17 studies that used HLM as their meta-analytic technique, 15 used MLE/REML. Two studies (i.e., Moeyaert et al., 2013b; Manolov et al., 2014) did not specify the estimation procedures they used. The remaining study (i.e., Idleman, 1993) used the sample average of the effect sizes as the aggregate treatment effect; the data analysis estimation methods were varied based on how the effect sizes (i.e., PND, Glass' delta, and Hedges' g) were calculated. Thus, the data-analysis estimation method of this study was coded as others.

### Design conditions

SCED meta-analytic data in Education and Psychology fields were generated under a variety of design conditions. This allows for investigating the appropriateness of the analysis model under various realistic conditions, allowing for more generalized conclusions. The first factor influencing the design condition is the number of units. The second factor pertains to the number of parameters (and values assigned to these parameters) used in the data generation model.

#### Number of units

The first factor influencing the design condition was the number of units at the observation level (repeated observations within participants), participant level (number of participants in each SCED study), and study level (number of studies in the meta-analysis). An overview of the number of units at the three levels is presented in this section, and more details can be found in Appendix C. The most often used value for the number of studies was 10 (88.9%; *n* = 16), followed by 30 (72.2%; *n* = 13). Regarding the number of participants, 72.2% *(n* = 13) included four participants, and 50% (*n* = 9) of the studies included five participants. When setting the number of observation sessions, 20 (58.3; *n* = 15) was the most commonly used value, followed by 10 (61.1%; *n* = 11) and 40 (44.4%; *n* = 8) measurement occasions. The most commonly used design condition related to the number of units was 10 studies with four participants within each primary study and 10 measurement occasions within each participant. Over half of the reports (55.6%; *n*=10) used this design condition. The second most commonly used conditions contained 10 studies, with seven participants for each primary study and 10 measurement occasions within each participant (i.e., 38.8%; *n* = 7).

#### Parameters in data generation model

The second factor influencing the design condition was the number of parameters and the values assigned to these parameters in the data generation model. The parameter values for studies using three-level HLM with four parameters (*n* = 14) are discussed first, followed by the two studies that used three-level HLM with two parameters. Lastly, the parameter values of the study by Idleman (1993) and Declercq et al. (2020), using other generation models, are presented.

#### Three-level HLM with four parameters

As discussed previously, 14 studies used HLM with four parameters as their data generation model. These studies included four fixed-effect parameters: the baseline level, baseline trend, the level and trend change between baseline, and intervention effects (i.e., γ_000_, γ_100_, γ_200_ and γ_300_, respectively; see Equations 4–6). The majority set the parameter value of the baseline level to zero (*n* = 9) or did not mention it (*n* = 3). Baek et al. (2020) set the baseline level at 70, and Manolov et al. (2014) set the baseline level at 7 or 40.

The average level change between baseline and intervention phases (i.e., γ_200_), which represents the intervention effect, was most often set to a value of 2 (*n* = 9), followed by 0 (*n* = 6) and 1 (*n* = 3). Eight studies included more than two conditions for the intervention effect. Five studies (i.e., Moeyaert et al., 2013a,b, 2014, 2016; Declercq et al., 2020) set the intervention effect as 0 or 2; Jamshidi et al. (2020) set the intervention effect at 0.2 or 2; Joo et al. (2021) set the intervention effect at 0.5 or 1; and Tsai (2011) set the intervention effect to 0, 0.5, or 1.

For the time trend in baseline data (i.e., γ_100_), nine out of the 14 studies set this parameter to zero. When researchers did include a trend change, time trends were set at 0.2, 04, 06, 1, 2, and 3. Two studies (i.e., Tsai, 2011; Ugille et al., 2012) had two or more values for the time trend. Expressly, Ugille et al. (2012) set the time trend in the baseline as either 0 or 2. Tsai (2011) set the time trend as 0.2, 0.4, or 0.6. Some studies (3 out of 14) did not mention the time trend in the baseline.

For the trend change parameter (γ_300_), 0.2 and 0 (*n* = 9 for each value) were the most often used values. Unlike the time trend in the baseline phase, quite a few studies (*n* = 11) included two conditions for trend change. Five studies (i.e., Ugille et al., 2012; Moeyaert et al., 2013a,b, 2014; Petit-Bois, 2014) kept the trend change as either zero or 0.2. Idleman (1993), Tsai (2011), Declercq et al. (2020), and Joo et al. (2021), and kept the trend change as zero. They also set the trend change as 2, 0.3, 0.25, and 0.4, respectively. For the remaining two studies that included two conditions for trend change, Jamshidi et al. (2020) set the trend change as either 0.2 or 2. Manolov et al. (2014) kept the trend change as either 1 or 3. One study (i.e., Baek et al., 2020) did not mention the value for the trend change between the baseline and intervention phases.

Many studies did not specify their parameter values for random effects (i.e., within-participant, between-participant, and between-study variance). Among the studies that did specify the within-participant variance, the most often used within-participant variance parameter value was 1 (*n* = 8). Researchers often kept the variance of the intervention effect the same as the variance of the baseline and the variance of the baseline time trend the same as the trend change. The most often used values of the between-participant variance of the baseline and intervention effect were 0.5 (*n* = 9) and 2 (*n* = 9), followed by 8 (*n* = 4). For between-participant variance of the time trend and trend change, the most often used parameter values were 0.05 and 0.2 (*n* = 8), followed by 0.08 (*n* = 4). When setting the between-study variance of the baseline level, 0.5 (*n* = 9), 2 (*n* = 7), and 8 (*n* = 4) were often used. The values 0.5 (*n* = 9), 2 (*n* = 8), and 8 (*n*=4) were often used for the between-study variance of the intervention effect. The values 0.05 (*n*=8), 0.2 (*n*=7), and 0.08 (*n* = 4) were often used for the between-study variance of the time trend in the baseline. Lastly, 0.05 (*n* = 9), 0.2 (*n* = 8), and 0.08 (*n* = 4) were often used for the between-study trend change between the baseline and intervention phases.

The between-participant and between-study covariances were often not modeled (*n* = 8). For studies that did specify the covariance, most studies set the covariance to 0 (*n* = 6). Like covariance, most studies did not specify autocorrelation (*n* = 6). For studies that did specify autocorrelation, most studies set the autocorrelation as 0 (*n* = 7) or 0.2 (*n* = 4).

#### Three-level HLM with two parameters

Two studies (Owens, 2011; Moeyaert et al., 2016) used three-level HLM with two parameters to generate SCED meta-analytic data. These studies included two fixed-effect parameters: the average baseline level and the level change between baseline and intervention level (i.e., γ_000_, γ_100_; see Equations 1–3). Moeyaert et al. (2016) kept the average baseline level at zero and set the level change between baseline and intervention at 0 or 2. Owens (2011) kept both fixed effects as 1.

In terms of random effects, Moeyaert et al. (2016) kept the within-participant variance at 1 and set the between-participant variances of baseline level and level change at 2 or 8. The between-study variance of baseline level and level change was also set at 2 or 8. Moeyaert et al. (2016) included two simulation studies. One kept the between-participant covariances and between-study covariances at zero. The other one set the between-participant and between-study covariances at −0.6 or −1.4 when the between-participant and between-study variances were 2, and −2.4 or −5.6 when the between-participant and between-study variances were 8. Moeyaert et al. (2016) focused on the covariance structure and did not include autocorrelation. Owens (2011) also kept the within-participant variance at 1. The between-participant variance of baseline level and level change were set at 2 or 0.2. The between-study variances of baseline level and level change were set at 0.05 or 0.5. Both between-participant and between-study covariances were kept at zero. Owens (2011) included autocorrelation and set the values at 0, 0.2, or 0.4.

#### Other meta-analytic models

Declercq et al. (2020) compared HLM with two parameters, four parameters, and six parameters. Idleman (1993) used simple average of effect sizes to synthesize effect sizes across studies and generated normal or chi-squared distributed data with and without autocorrelation. When the data followed a normal distribution, the baseline level was generated around 5, and the intervention effect was generated as 3.3, 2.2, or 1.5. When the data followed a chi-squared distribution, the baseline level was 5, while the intervention effect was 4.24, 6.23, or 9.33.

### Statistical properties per meta-analytic technique

An overview of the statistical properties investigated by the 18 included SCED meta-analysis reports is displayed in Appendix C. Among the 17 studies that used HLM as their meta-analytic method, 15 studied confidence interval coverage (CI). CI was the most often studied statistical property, followed by bias (*n* = 14) and relative bias (*n* = 13). More than half of the included reports (*n* = 11) also investigated standard error (SE). MSE was also investigated by a large proportion of meta-analytic studies (n = 11). Other statistical properties such as Power (*n* = 6), Type I error (*n* = 6), relative SE bias (*n* = 5), and RMSE (*n* = 4) were also investigated by some studies. One thing worth mentioning is that Manolov et al. (2014) did not study any statistical properties. The possible reason is that Manolov et al. (2014) focused on the weighting stages instead of evaluating the statistical properties of parameter estimates. The majority of studies investigated both Power and Type I error together. However, Moeyaert et al. (2013a) reported Power but not Type I error. Declercq et al. (2020) studied Type I error rate but not Power.

### Research purpose and recommendations

The most commonly reported purpose was to examine the performance or the appropriateness of the model(s) in terms of their statistical properties (*n* = 5) and handling data complexity (*n* = 5). Data complexities included but were not limited to heterogeneity, autocorrelation, and dependent effect sizes. Handling model misspecification was next, investigated by three studies. Two studies (i.e., Moeyaert et al., 2013b; Declercq et al., 2020) examined the performance/appropriateness of the model(s) in handling different SCED designs, and two (i.e., Idleman, 1993; Ugille et al., 2012) looked at different effect size metrics. Idleman (1993) compared the appropriateness of PND, Glass' effect sizes, and Hedge's unbiased estimators for single-case research. Ugille et al. (2012) compared the performance of unstandardized and standardized regression coefficients as the effect size metrics in SCED meta-analysis. Although most included studies focused on the meta-analysis techniques and models, two studies focused on some other statistical techniques. Specifically, one study (i.e., Manolov et al., 2014) compared several weighting strategies, and another study (i.e., Ugille et al., 2014) explored four bias correction approaches for HLM meta-analysis of single-case studies with four parameters. An overview of the specific studies per research purpose is presented in Table 2.

**Table 2:** Research purpose per study.

**Purpose**	***n* (%)**	**References**
Examining the performance/appropriateness of a model or comparing the performances/appropriateness of multiple models in terms of statistical properties.	5 (28%)	Owens, 2011; Tsai, 2011; Moeyaert et al., 2013a; Jamshidi et al., 2021; Joo et al., 2021
Examining the performance/appropriateness of a model or comparing the performances/appropriateness of multiple models in handling data complexity.	5 (28%)	Moeyaert et al., 2014; Petit-Bois, 2014; Joo et al., 2019; Baek et al., 2020; Jamshidi et al., 2020
Examining the performance/appropriateness of a model or comparing the performances/appropriateness of multiple models in handling model misspecification.	3 (17%)	Petit-Bois, 2014; Moeyaert et al., 2016; Petit-Bois et al., 2016
Examining the performance/appropriateness of a model or comparing the performances/appropriateness of multiple models in handling different SCED designs.	2 (11%)	Moeyaert et al., 2013b; Declercq et al., 2020
Examining the performance/appropriateness of a model or comparing the performances/appropriateness of multiple models with different effect size metrics.	2 (11%)	Idleman, 1993; Ugille et al., 2012
Others	2 (11%)	Manolov et al., 2014; Ugille et al., 2014

Some of the included studies made recommendations about the number of studies and observations to use. Moeyaert et al. (2013a, 2014, 2016) recommended using a set of homogeneous primary studies (i.e., low between-study variance). The number of primary studies should be more than 30, and the number of observations should be at least 30 to reach a reasonable power. Jamshidi et al. (2020) encouraged including more than 40 studies in a meta-analysis, especially when the research interest lies in estimating the between-study variance. Tsai (2011) recommended to use 50 studies in a meta-analysis of SCEDs unless the expected intervention effect is large. Ugille et al. (2012) suggested including 20 or more observation sessions per participant when using standardized effect sizes in the multilevel meta-analysis. Lastly, Jamshidi et al. (2021) recommended using more than 10 observation sessions, especially when estimating the between-participant and between-study variance.

## Discussion

This study provides an overview of empirically validated meta-analytic techniques for synthesizing SCED studies in Education and Psychology fields. The findings of this systematic research can be helpful for methodologists in Education and Psychology fields to further develop the methodology of SCED meta-analytic techniques. Additionally, applied researchers and research synthesists in Education and Psychology fields can gain insight into the conditions under which the studied meta-analytic techniques for SCED studies are appropriate and recommended.

This systematic review results show that three-level HLM approach is the most commonly empirically validated meta-analytic technique. Using three-level HLM is reasonable as a hierarchical data structure characterizes SCED meta-analysis data: repeated observations (i.e., level 1) are nested within participants (i.e., level 2), and participants, in turn, are nested within studies (i.e., level 3). HLM can take this clustered data structure into account (Van den Noortgate and Onghena, 2003a,b, 2008), making it the most promising meta-analytic technique for SCED studies. Other benefits of using HLM are robustness in handling complex data hierarchies, and enhanced sensitivity to detect treatment effects (Price et al., 2008; McNeish et al., 2017). These benefits are often of paramount importance in SCED research.

For any research and statistical technique to be applied and recommended, sufficient methodological work is needed to investigate and empirically validate it. In addition, methodologists should ensure the technique has good statistical properties, such as providing unbiased estimation, and can perform well with complex data due to robustness against data complexity. Monte Carlo simulation study is a common way to examine the techniques' statistical properties. These simulation studies enable methodologists to systematically explore the technique's behavior under various conditions, including different sample sizes, effect sizes, and data distributions, helping researchers gain insights into the robustness and suitability of the used technique for practical application.

Concerning the research purpose, when the studies aimed to examine the performance of HLM, most simulation studies directly adapted three-level HLM, including four parameters as proposed by Van den Noortgate and Onghena (2003b, 2008) to generate SCED data across studies. A couple of simulation studies used the multilevel models proposed by Huitema and McKean (1991, 2000, 2007). The difference between these two models is how they estimate the trend change between the baseline and intervention phases. However, these two models have the same parameters and estimate the same number of fixed parameters. Both models included phase (i.e., a dummy variable with 0 = baseline and 1 = intervention phase), time, and the interaction between time and phase in their model. They estimate the baseline level, time trend, level change and trend change between baseline and intervention. Some researchers have extended these models by adding additional variables. For example, Moeyaert et al. (2014) proposed an extended model with an additional dummy-coded variable to control for external event effects. These extensions reflect the dynamic nature of SCED research and the necessity of considering external factors that may influence outcomes.

Most studies used HLM as the meta-analytic simulated SCED data with MBD. Using MBD is reasonable as the MBD is the most commonly used design in the SCED field (Hammond and Gast, 2010; Shadish and Sullivan, 2011). The remaining studies used either AB or repeated AB designs (i.e., Idleman, 1993; Coleman, 2006; Owens, 2011; Tsai, 2011; Declercq et al., 2020). One possible reason is that meta-analysis of MBD data across multiple participants and repeated AB design data can directly adapt the models proposed by Van den Noortgate and Onghena (2003b, 2008). Meta-analysis of other SCED design data, such as ATD, requires model modification, which is not yet developed. This raises important questions about the generalizability of HLM to various SCED designs and underscores the need for future research to address these gaps in modeling strategies.

Most studies included more than one design condition based on the number of units (i.e., the number of studies, participants, and measurements), the number of parameters, and the values assigned to the parameters. The most commonly used design condition includes 10 studies with four participants for each study and 10 observation sessions for each participant. Some extreme conditions, such as 80 studies and 50 participants per study, were also investigated by some methodological studies. These unrealistic design conditions were often simulated as an ideal situation to test the model. In terms of the parameters, the baseline level, time trend, and trend change between baseline and intervention were often set at zero, whereas the level change between baseline and intervention was often set at 1 or 2. Researchers often use the level change between the baseline and intervention phase as an indicator of the intervention effect, making it the parameter of interest in a SCED study. This is the possible reason why studies often set a level change for the simulated data while keeping the other parameter values constant. For random effects such as between-participant, between-study, and within-participant variance, the hypothesized values were selected to reflect the design conditions in practice. The variability in the design conditions used in SCED studies suggests that researchers should carefully consider and justify their choice of design conditions.

A large number of primary studies (more than 30) and observation sessions (more than 20) per participant are recommended. Some studies made recommendations about which conditions for a specific model work best. For example, Coleman (2006) recommended that researchers include 20 units in level one (e.g., 20 measurements) and 30 units in level two (e.g., 30 participants) for a meta-analysis to have enough power. Ugille et al. (2012) recommended including 20 or more observations when conducting a multilevel meta-analysis of SCEDs. Moeyaert et al. (2013a, 2014) also recommended that researchers include 30 or more studies and 20 or more observations within participants to have sufficient power to detect the intervention effect. Ugille et al. (2012) and Moeyaert et al. (2014) both mentioned the importance of using a homogeneous set of studies (i.e., small between-study variance). Jamshidi et al. (2020, 2021) recommended having 40 or more studies to better estimate between-study variance. A critical assessment of these suggestions reveals their potential influence on the accuracy and reliability of between-study variance estimation. It is pertinent to consider whether these conditions can be met in diverse research context.

Most methodological studies not only considered the baseline level and the level change (between baseline and intervention phases) of the SCED data but also considered the baseline trend and trend change. However, some studies, such as Owens (2011) and Moeyaert et al. (2016), only considered the baseline level and the level change. One possible reason is that the three-level HLM with two parameters has fewer parameters, which is a good starting point to examine the appropriateness of the three-level HLM. For example, Moeyaert et al. (2016) focused on the misspecifications of the covariance structure. Thus, Moeyaert et al. (2016) used a three-level HLM with two parameters, which reduces the number of fixed parameters that need to be estimated to isolate and simplify the situation. A critical analysis of this choice should explore the potential implications for model fit and the validity of conclusions drawn from the simplified model.

In addition, researchers in the field recommend bootstrapping or Bayesian approaches to reduce parameter bias. For example, Rindskopf (2014) argues that despite the multilevel structure of single-case data, Bayesian methods are more useful than simple linear models due to several reasons: taking into account uncertainty in random effects when estimating fixed effects, being able to fit complex models that represent accurately the behavior being modeled; more accurately estimating groups of parameters using shrinkage methods; including prior information; and stating more straightforward interpretation. These approaches are particularly well-suited to address the unique challenges and complexities associated with single-case data. However, researchers in the field should carefully assess the applicability of these methods to their specific research contexts while recognizing that context-specific considerations may influence the choice of statistical approach.

Especially, Declercq et al. (2019) compared LMM (also known as HLM, multilevel analysis) with GLMM (Generalized linear mixed model) using REML as the estimation method in their simulation. They found that GLMM performed better regarding the goodness of fit and power. However, in estimating the average effect size across studies, the simple LMM works equally well with GLMM, as the parameter recovery is the same under both models. Furthermore, they assumed the outcomes followed a Poisson or a normal distribution. With transformation, Declercq et al. (2019) claimed that the average baseline response was 2, 4, 20, or 30, and the average treatment effect was 1, 1.5, 1.6, or 3.5. Shadish et al. (2013) expanded the HLM framework for synthesizing SCED effect sizes across studies by proposing a generalized multilevel analysis (i.e., generalized linear mixed model). The generalized linear mixed model (GLMM) is a combination of linear mixed modeling (LMM) and generalized linear modeling (GLM), taking both the hierarchical data structure and count data into account (Declercq et al., 2019). Furthermore, besides supporting a non-continuous data format, it supports dependent variables that are not normally distributed (Garson, 2013).

It is worth noting that Coleman (2006) and Declercq et al. (2019) studied two-level HLM but called their study a multilevel meta-analysis. These studies synthesized SCED data across participants within one study. Chen and Chen's (2014) study, which was excluded from the current systematic review, investigated combining n-of-1 trials and considered each subject an *n*-of-1 trial. Consequently, it is debatable if these studies can be classified as meta-analyses as they do not combine research evidence across studies, which is the original meaning of *meta-analysis* (Glass, 2012).

## Limitations

Like any systematic review, this systematic review has its limitations. The search was done in December 2021, and it has been more than a year during which new studies may have been published. This means that the results of this review may reflect less up-to-date evidence.

Moreover, We acknowledge the limitation imposed by the relatively small number of studies included in this systematic review, comprising a total of 18 reports. This constraint naturally affects the generality and level of detail in the conclusions drawn from our analysis. While we have made every effort to extract meaningful insights from this limited pool of studies, we recognize that our findings must be considered within the context of this constraint. The limited number of studies does not allow for highly detailed claims. As such, our conclusions should be understood as reflective of the available evidence rather than comprehensive or all-encompassing. We have, however, strived to mitigate this limitation by employing rigorous systematic review methods, including well-defined inclusion and exclusion criteria. We view this review as a foundational exploration of the topic, intended to guide future research endeavors and offer initial insights into the subject matter.

It is important to note that we purposely narrowed down the scope to the field of Education and Psychology, guided by the principles of feasibility, interest, novelty, ethics, and relevance outlined in the FINER criteria (Cummings et al., 2013). This strategic focus enhances the applicability of our findings to these specific domains. Given the exponential increase in SCED meta-analyses, narrowing our focus to Education and Psychology makes our systematic review not only feasible but also highly relevant within these fields. Moreover, this systematic review underscores the need for a separate systematic review tailored to other fields such as medicine, recognizing the distinct challenges and requirements that each domain presents.

## Data availability statement

The original contributions presented in the study are included in the article/Supplementary material, further inquiries can be directed to the corresponding author.

## Author contributions

MM: conceptualization, interpretation results, write-up, and supervision. XX, MD-C, and PY: systematic search, analysis, and write-up. All authors contributed to the article and approved the submitted version.

## References

[B1] Asaro-Saddler K. Moeyaert M. Xu X. Yerden X. (2021). Multilevel meta-analysis of the effectiveness of self-regulated strategy development in writing for children with ASD. Exceptionality 29, 150–166. 10.1080/09362835.2020.1850457

[B2] ^*^ Baek E. Luo W. Henri M. (2020). Issues and solutions in meta-analysis of single-case design with multiple dependent variables using multilevel modeling. J. Exp. Educ. 10.1080/00220973.2020.182134236253594

[B3] Borenstein M. Cooper H. Hedges L. Valentine J. (2009). Effect sizes for continuous data. Handb. Res. Synth. Meta Anal. 2, 221–235.

[B4] Caron E. B. Dozier M. (2019). Effects of fidelity-focused consultation on clinicians' implementation: an exploratory multiple baseline design. Administr. Policy Mental Health Serv. Res. 46, 445–457. 10.1007/s10488-019-00924-3 30783903 PMC7161185

[B5] Center B. A. Skiba R. J. Casey A. (1985). A methodology for the quantitative synthesis of intra-subject design research. J. Spec. Educ. 19, 387–400.

[B6] Chen M. Pustejovsky J. E. (2022). Multi-level meta-analysis of single-case experimental designs using robust variance estimation. Psychol. Methods. 10.1037/met0000510 35786985

[B7] Chen X. Chen P. (2014). A comparison of four methods for the analysis of N-of-1 trials. PLoS ONE 9, e87752. 10.1371/journal.pone.0087752 24503561 PMC3913644

[B8] Cohen J. (2013). Statistical Power Analysis for the Behavioral Sciences. New York, NY: Accademic Press.

[B9] Coleman J. L. (2006). A Simulation Study of the Piecewise Hierarchical Model Approach to Meta-analysis of Single-Subject Data, Vol. 68. ProQuest Dissertations Publishing.

[B10] Cummings S. R. Browner W. S. Hulley S. B. (2013). Conceiving the research question and developing the study plan. Design. Clin. Res. 4, 14–22.

[B11] ^*^ Declercq L. Jamshidi L. Fernandez Castilla B. Moeyaert M. Beretvas S. N. Ferron J. M. . (2020). Multilevel meta-analysis of individual participant data of single-case experimental designs: One-stage versus two-stage methods. Multivar. Behav. Res. 10.1080/00273171.2020.1822148 32996335

[B12] Declercq L. Jamshidi L. Fernández-Castilla B. Beretvas S. N. Moeyaert M. Ferron J. M. . (2019). Analysis of single-case experimental count data using the linear mixed effects model: a simulation study. Behav. Res. Methods 51, 2477–2497. 10.3758/s13428-018-1091-y 30105444

[B13] Everitt B. S. Howell D. C. (2021). Encyclopedia of Statistics in Behavioral Science–Vol. 2. Chichester: Wiley.

[B14] Fingerhut J. Moeyaert M. (2022). Training individuals to implement discrete trials with fidelity: a meta-analysis. Focus Autism Other Dev. Disabl. 37, 239–250. 10.1177/10883576221081076

[B15] Garson G. D. (2013). Fundamentals of hierarchical linear and multilevel modeling. Hierarch. Linear Model. Guide Appl. 3–25. 10.4135/9781483384450.n1

[B16] Glass G. V. (2012). Meta-analysis: the quantitative synthesis of research findings. In: Handbook of Complementary Methods in Education Research, eds J. L. Green, J. L. Green, G. Camilli, P. B. Elmore, and P. B. Elmore (Lawrence Erlbaum Associates, Inc.), 427–438.

[B17] Hammond D. Gast D. L. (2010). Descriptive analysis of single subject research designs: 1983–2007. Educ. Train. Autism Dev. Disabil. 45, 187–202. Available online at: https://www.jstor.org/stable/23879806

[B18] Higgins J. P. T. Thomas J. Chandler J. Cumpston M. Li T. Page M. J. . (2021). Handbook for Systematic Reviews of Interventions, Version 6.2.

[B19] Huitema B. E. McKean J. W. (1991). Autocorrelation estimation and inference with small samples. Psychol. Bull. 110, 291–304. 10.1037/0033-2909.110.2.291

[B20] Huitema B. E. McKean J. W. (2000). Design specification issues in time-series intervention models. Educ. Psychol. Meas. 60, 38–58. 10.1177/00131640021970358

[B21] Huitema B. E. McKean J. W. (2007). Identifying autocorrelation generated by various error processes in interrupted time-series regression designs: a comparison of AR1 and portmanteau tests. Educ. Psychol. Meas. 67, 447–459. 10.1177/0013164406294774

[B22] ^*^ Idleman L. S. (1993). The Comparison of Three Meta-Analytic Metrics for Single-Subject Research. ProQuest Dissertations Publishing, Georgia State University.

[B23] ^*^ Jamshidi L. Declercq L. Fernández-Castilla B. Ferron J. M. Moeyaert M. Beretvas S. N. . (2020). Multilevel meta-analysis of multiple regression coefficients from single-case experimental studies. Behav. Res. Methods 52, 2008–2019. 10.3758/s13428-020-01380-w 32144730

[B24] ^*^ Jamshidi L. Declercq L. Fernández-Castilla B. Ferron J. M. Moeyaert M. Beretvas S. N. . (2021). Bias adjustment in multilevel meta-analysis of standardized single-case experimental data. J. Exp. Educ. 89, 344–361. 10.1080/00220973.2019.1658568

[B25] Jamshidi L. Heyvaert M. Declercq L. Fernández-Castilla B. Ferron J. M. Moeyaert M. . (2022). A systematic review of single-case experimental design meta-analyses: characteristics of study designs, data, and analyses. Evid. Based Commun. Assess. Intervent. 1–25. 10.1080/17489539.2022.2089334

[B26] ^*^ Joo S. H. Ferron J. M. Moeyaert M. Beretvas S. N. Van den Noortgate W. (2019). Approaches for specifying the level-1 error structure when synthesizing single-case data. J. Exp. Educ. 87, 55–74. 10.1080/00220973.2017.1409181

[B27] ^*^ Joo S. H. Wang Y. Ferron J. Beretvas S. N. Moeyaert M. Van Den Noortgate W. (2021). Comparison of within-and between-series effect estimates in the meta-analysis of multiple baseline studies. J. Educ. Behav. Stat. 47, 131–166.

[B28] ^*^ Manolov R. Guilera G. Sierra V. (2014). Weighting strategies in the meta-analysis of single-case studies. Behav. Res. Methods 46, 1152–1166. 10.3758/s13428-013-0440-0 24488814

[B29] McNeish D. Stapleton L. M. Silverman R. D. (2017). On the unnecessary ubiquity of hierarchical linear modeling. Psychol. Methods 22, 114. 10.1037/met0000078 27149401

[B30] Microsoft Corporation (2018). Microsoft Excel. Retrieved from: https://office.microsoft.com/excel (accessed January 2023).

[B31] Moeyaert M. (2019). Quantitative synthesis of research evidence: multilevel meta-analysis. Behav. Disord. 44, 241–256. 10.1177/0198742918806926

[B32] ^*^ Moeyaert M. Ugille M. Ferron J. M. Beretvas S. N. den Noortgate W. V. (2014). Three-level analysis of single-case experimental data: empirical validation. J. Exp. Educ. 82, 1–21. 10.1080/00220973.2012.745470

[B33] ^*^ Moeyaert M. Ugille M. Ferron J. M. Beretvas S. N. Van den Noortgate W. (2013a). The three-level synthesis of standardized single-subject experimental data: a Monte Carlo simulation study. Multivariate Behav. Res. 48, 719–748. 10.1080/00273171.2013.816621 26741060

[B34] ^*^ Moeyaert M. Ugille M. Ferron J. M. Beretvas S. N. Van den Noortgate W. (2013b). Modeling external events in the three-level analysis of multiple-baseline across-participants designs: a simulation study. Behav. Res. Methods 45, 547–559. 10.3758/s13428-012-0274-1 23233188

[B35] ^*^ Moeyaert M. Ugille M. Ferron J. M. Beretvas S. N. Van den Noortgate W. (2016). The misspecification of the covariance structures in multilevel models for single-case data: a Monte Carlo simulation study. J. Exp. Educ. 84, 473–509. 10.1080/00220973.2015.1065216

[B36] Moeyaert M. Ugille M. Ferron J. M. Onghena P. Heyvaert M. Beretvas S. N. . (2015). Estimating intervention effects across different types of single-subject experimental designs: empirical illustration. Sch. Psychol. Q. 30, 50. 10.1037/spq0000068 24884449

[B37] Moeyaert M. Yang P. Xu X. (2022). The power to explain variability in intervention effectiveness in single-case research using hierarchical linear modeling. Perspect. Behav. Sci. 45, 13–35 10.1007/s40614-021-00304-z 35342874 PMC8894540

[B38] Moeyaert M. Yang P. Xu X. Kim E. (2021). Characteristics of moderators in meta-analyses of single-case experimental design studies. Behav. Modif. 10.1177/01454455211002111 33759586

[B39] Myung I. J. (2003). Tutorial on maximum likelihood estimation. J. Math. Psychol. 47, 90–100. 10.1016/S0022-2496(02)00028-7

[B40] Onghena P. (2005). “Single-case designs,” in Encyclopedia of Statistics in Behavioral Science. Vol. 4, ed B. E. D. Howell (Chichester: Wiley), 1850–1854.

[B41] ^*^ Owens C. M. (2011). Meta-Analysis of Single-Case Data: A Monte Carlo Investigation of a Three Level Model. ProQuest Dissertations Publishing, University of South Florida.

[B42] Page M. J. McKenzie J. E. Bossuyt P. M. Boutron I. Hoffmann T. C. Mulrow C. D. . (2021). The PRISMA 2020 statement: an updated guideline for reporting systematic reviews. Syst. Rev. 10, 1–11. 10.1186/s13643-021-01626-4 33781348 PMC8008539

[B43] Pan J. X. Fang K. T. (2002). “Maximum likelihood estimation,” in Growth Curve Models and Statistical Diagnostics (New York, NY: Springer), 77–158.

[B44] ^*^ Petit-Bois M. (2014). A Monte Carlo Study: The Consequences of the Misspecification of the Level-1 Error Structure when Meta-Analyzing Single-Case Designs. ProQuest Dissertations Publishing, University of South Florida.

[B45] ^*^ Petit-Bois M. Baek E. K. Van den Noortgate W. Beretvas S. N. Ferron J. M. (2016). The consequences of modeling autocorrelation when synthesizing single-case studies using a three-level model. Behav. Res. Methods 48, 803–812. 10.3758/s13428-015-0612-1 26463724

[B46] Petrocchi N. Cosentino T. Pellegrini V. Femia G. D'Innocenzo A. Mancini F. (2021). Compassion-focused group therapy for treatment-resistant OCD: initial evaluation using a multiple baseline design. Front. Psychol. 11, 594277. 10.3389/fpsyg.2020.594277 33510677 PMC7835278

[B47] Price M. Anderson P. Henrich C. C. Rothbaum B. O. (2008). Greater expectations: Using hierarchical linear modeling to examine expectancy for treatment outcome as a predictor of treatment response. Behav. Ther. 39, 398–405. 10.1016/j.beth.2007.12.002 19027436 PMC3678533

[B48] Quzzani M. Hammady H. Fedorowicz Z. Elmagarmid A. (2016). Rayyan — a web and mobile app for systematic reviews. Syst. Rev. 5, 210. 10.1186/s13643-016-0384-4 PMC5139140 27919275

[B49] Rindskopf D. (2014). Nonlinear Bayesian analysis for single case designs. J. Sch. Psychol. 52, 179–189. 10.1016/j.jsp.2013.12.003 24606974

[B50] Rosenbaum P. R. Everitt B. S. Howell D. (2005). Encyclopedia of Statistics in Behavioral Science. New York, NY: Wiley.

[B51] Rossi R. J. (2018). Mathematical Statistics: An Introduction to Likelihood Based Inference. Hoboken, NJ: John Wiley and Sons.

[B52] Shadish W. R. Kyse E. N. Rindskopf D. M. (2013). Analyzing data from single-case designs using multilevel models: new applications and some agenda items for future research. Psychol. Methods 18, 385. 10.1037/a0032964 23834421

[B53] Shadish W. R. Sullivan K. J. (2011). Characteristics of single-case designs used to assess intervention effects in 2008. Behav. Res. Methods 43, 971–980. 10.3758/s13428-011-0111-y 21656107

[B54] ^*^ Tsai S. P. (2011). Using Multilevel Modeling in Synthesizing Single-Subject Research Data With Trend—A Monte Carlo Study. ProQuest Dissertations Publishing, University of Washington.

[B55] ^*^ Ugille M. Moeyaert M. Beretvas S. N. Ferron J. Van den Noortgate W. (2012). Multilevel meta-analysis of single-subject experimental designs: a simulation study. Behav. Res. Methods 44, 1244–1254. 10.3758/s13428-012-0213-1 22648696

[B56] ^*^ Ugille M. Moeyaert M. Beretvas S. N. Ferron J. M. Van den Noortgate W. (2014). Bias corrections for standardized effect size estimates used with single-subject experimental designs. J. Exp. Educ. 82, 358–374. 10.1080/00220973.2013.813366

[B57] Van den Noortgate W. Onghena P. (2003a). A parametric bootstrap version of Hedges' homogeneity test. J. Mod. Appl. Stat. Methods 2, 7. 10.22237/jmasm/1051747620

[B58] Van den Noortgate W. Onghena P. (2003b). Combining single-case experimental data using hierarchical linear models. Sch. Psychol. Q. 18, 325. 10.1521/scpq.18.3.325.22577

[B59] Van den Noortgate W. Onghena P. (2007). The aggregation of single-case results using hierarchical linear models. Behav. Anal. Today 8, 196. 10.1037/h0100613

[B60] Van den Noortgate W. Onghena P. (2008). A multilevel meta-analysis of single-subject experimental design studies. Evid. Based Commun. Assess. Interv. 2, 142–151 10.1080/1748953080250536222648696

