# Supplementary material for: Single-case design meta-analyses in education and psychology: a systematic review of methodology
Source: Front Res Metr Anal. 2023 Nov 13;8:1190362. doi: 10.3389/frma.2023.1190362 (PMC10679716; doi:10.3389/frma.2023.1190362)
Supplement: Supplementary file 2 [file Data_Sheet_2.pdf]

Appendix A

The search terms were searched “Anywhere” (in PsychINFO and ERIC), “All fields” (in WOS) and “Abstract” (in ProQuest Dissertations & Theses Global). The syntax for the search in these databases was as follow for the three rows used in Advanced Search for PsychINFO, ERIC, and ProQuest Dissertations & Theses Global and Basic Search in WOS : for the first row we used ("single case" OR "single subject" OR "N of 1" OR "small N" OR "multiple baseline" OR "alternating treatment" OR "reversal design" OR "withdrawal design"), for the second row we used ("meta-analysis" OR "synthesis" OR "review"), and for the first row we used (“simulation" OR "Monte Carlo" OR "monte carlo”). Each row was connected by AND.

In DANS EASY Archive and PubMed, we search the whole string together as: (("single case" OR "single subject" OR "N of 1" OR "small N" OR "multiple baseline" OR "alternating treatment" OR "reversal design" OR "withdrawal design") AND ("meta-analysis" OR "synthesis" OR "review") AND (“simulation" OR "Monte Carlo" OR "monte carlo”)).

Appendix B

**Meta-Analytic Techniques**	1= Three-level hierarchical linear modeling (HLM or multilevel analysis, linear mixed effects model)<br>2= Generalized linear mixed model (GLMM)<br>3= Meta-analysis of summary data (including the simple average of effect sizes, the median of effect sizes, and weighted average of effect sizes)<br>4= Other(s)
**Data Generation Models**	1= Three-level HLM with two parameters<br>Level 1: $y_{ijk} = \beta_{0jk} + \beta_{1jk}Phase_{ijk} + e_{ijk}$ (1)<br>Level 2: $\left\{\beta_{0jk} = \theta_{00k} + u_{0jk}\right\}$ (2)<br>$\left\{\beta_{1jk} = \theta_{10k} + u_{1jk}\right\}$<br>Level 3: $\{\theta_{00k} = \gamma_{000} + v_{00k}\}$ (3)<br>$\{\theta_{10k} = \gamma_{100} + v_{10k}\}$<br><br>2= Three-level HLM with four parameters<br>Level 1: $y_{ijk} = \beta_{0jk} + \beta_{1jk}Phase_{ijk} + \beta_{2jk}Time0_{ijk} +$<br>$\beta_{3jk}PhaseTimeC_{ijk} + e_{ijk}$ (4)<br>Level 2: $\left\{\beta_{0jk} = \theta_{00k} + u_{0jk}\right\}$ (5)<br>$\left\{\beta_{1jk} = \theta_{10k} + u_{1jk}\right\}$<br>$\left\{\beta_{2jk} = \theta_{20k} + u_{2jk}\right\}$<br>$\left\{\beta_{3jk} = \theta_{30k} + u_{3jk}\right\}$<br>Level 3: $\{\theta_{00k} = \gamma_{000} + v_{00k}\}$ (6)<br>$\{\theta_{10k} = \gamma_{100} + v_{10k}\}$<br>$\{\theta_{20k} = \gamma_{200} + v_{20k}\}$<br>$\{\theta_{30k} = \gamma_{300} + v_{30k}\}$<br>3= Other(s)

**Data Analysis Models**	1= The same as Data Generation Model<br>2= Other model/ Different from Data Generation Model (provide more details in the parenthesis)
**Data Generation Designs**	1= MBD<br>2= AB<br>3= Replicated AB design<br>4= Random phase design<br>5= Other
**Data Analysis Estimation Methods**	1=MLE or REML<br>2=OLS<br>3=GLS<br>4=Other(s)
**Design Conditions**	Please specify the information about the number of units in each level (i.e., the number of primary SCED studies, the number of cases in each study, and the number of measurement occasions)<br><br>- The number of measurements occasions (nested within cases)<br>- The number of cases (nested within SCED studies)<br>- The number of studies (combined in the SCED meta-analytic model)
**Parameter Values**	For studies used *three-level HLM with four parameters* include: the average baseline level, baseline trend, the level and trend change between baseline and intervention effects (i.e., γ_{000} , γ_{100}, γ_{200} and γ_{300}, respectively; see equation 4-6) as fixed-effect parameters.<br><br>For random effects: within-case variance, between-case variances, and between-study variance, the between-case and between-study covariances, and autocorrelation should be coded.<br><br>For studies that used *three-level HLM with two parameters*, the fixed-effect parameters are: the average baseline level and the level change between baseline and intervention effects (i.e., γ_{000} , γ_{100}; see equation 1-3).<br>For random effects: between-study variances, within-case variance, between-case variances of baseline level and level change, between-case covariances and between-study covariances should be coded. Information about autocorrelation should be coded too.<br><br>For studies using *other data generation models,* information about autocorrelation, the baseline level, the baseline trend, the intervention or treatment effect, the level and trend change between baseline and intervention phases, the between-case and between study variances, the within-case variance should be coded if reported.

**Statistical Properties Investigated**	May include but not limited to the following list (please specify the ones investigated):<br>Bias (Absolute bias)<br>Relative (parameter) bias<br>Relative SE bias<br>Confidence interval (coverage)<br>MSE<br>Relative MSE<br>Root mean square error (RMSE)<br>SE<br>Type I error<br>Power<br>Between-study and between-case variances<br>Other (provide more details in the parenthesis)
**Research Purpose per Study**	May examine the performance/appropriateness of a model or compare the performances/appropriateness of multiple models<br>1) in terms of statistical properties<br>2) in handling different SCED designs<br>3) in handling data complexity<br>4) with different effect size metric<br>5) in handling model misspecification<br>6) others

Appendix C

Tables

Table 1

Overview of Units

Unit	Values	n (%)
Number of Studies	5	1 (5.6 %)
	7	2 (11.1%)
	**10**	16 (88.9%)
	14	1 (5.6%)
	20	2 (11.1%)
	**30**	13 (72.2%)
	40	2 (11.1%)
	50	1 (5.6%)
	60	1 (5.6%)
	80	1 (5.6%)
Number of participants	3	5 (27.8%)
	**4**	13 (72.2%)
	5	2 (11.1%)
	**7**	9 (50%)
	8	4 (22.2%)
	10	1 (5.6%)
Number of Measurement Occasions	**10**	11 (61.1%)
	15	3 (16.7%)
	**20**	15 (83.3%)
	25	2 (11.1%)
	28	1 (5.6%)
	30	5 (27.8%)
	40	8 (44.4%)
	Missing	1 (5.6%)

Table 2

Overview of Statistical Properties Per Meta-Analytic Techniques

Meta-analytic Techniques (number of studies)	Statistical Properties (n)								
	Bias	Relative (parameter) bias	Relative SE	SE	MSE	CI	Type I error	Power	RMSE
HLM (n = 17)	(14)	(13)	(5)	(11)	(9)	(15)	(6)	(6)	(4)
Simple Average of Effect Sizes (n = 1)	(1)	-	-	-	-	1	-	-	-
